# The Effects of the Mechanical Properties of Vascular Grafts and an Anisotropic Hyperelastic Aortic Model on Local Hemodynamics during Modified Blalock–Taussig Shunt Operation, Assessed Using FSI Simulation

**DOI:** 10.3390/ma15082719

**Published:** 2022-04-07

**Authors:** Alex G. Kuchumov, Aleksandr Khairulin, Marina Shmurak, Artem Porodikov, Andrey Merzlyakov

**Affiliations:** 1 Department of Computational Mathematics, Mechanics, and Biomechanics, Faculty of Applied Mathematics and Mechanics, Perm National Research Polytechnic University, 614990 Perm, Russia; s.xayrulin@mail.ru (A.K.); shmurak2007@yandex.ru (M.S.); 2 Federal Center of Cardiovascular Surgery, 614990 Perm, Russia; porodickov.a@yandex.ru; 3 Department of Continuum Mechanics and Computing Technologies, Faculty of Mechanics and Mathematics, Perm State National Research University, 614990 Perm, Russia; merzlyakov@psu.ru

**Keywords:** hemodynamics, modified Blalock–Taussig shunt, hyperelasticity, anisotropy, fluid–structure interaction

## Abstract

Cardiovascular surgery requires the use of state-of-the-art artificial materials. For example, microporous polytetrafluoroethylene grafts manufactured by Gore-Tex^®^ are used for the treatment of cyanotic heart defects (i.e., modified Blalock–Taussig shunt). Significant mortality during this palliative operation has led surgeons to adopt mathematical models to eliminate complications by performing fluid–solid interaction (FSI) simulations. To proceed with FSI modeling, it is necessary to know either the mechanical properties of the aorta and graft or the rheological properties of blood. The properties of the aorta and blood can be found in the literature, but there are no data about the mechanical properties of Gore-Tex^®^ grafts. Experimental studies were carried out on the mechanical properties vascular grafts adopted for modified pediatric Blalock–Taussig shunts. Parameters of two models (the five-parameter Mooney–Rivlin model and the three-parameter Yeoh model) were determined by uniaxial experimental curve fitting. The obtained data were used for patient-specific FSI modeling of local blood flow in the “aorta-modified Blalock–Taussig shunt–pulmonary artery” system in three different shunt locations: central, right, and left. The anisotropic model of the aortic material showed higher stress values at the peak moment of systole, which may be a key factor determining the strength characteristics of the aorta and pulmonary artery. Additionally, this mechanical parameter is important when installing a central shunt, since it is in the area of the central anastomosis that an increase in stress on the aortic wall is observed. According to computations, the anisotropic model shows smaller values for the displacements of both the aorta and the shunt, which in turn may affect the success of preoperative predictions. Thus, it can be concluded that the anisotropic properties of the aorta play an important role in preoperative modeling.

## 1. Introduction

The mortality rate of cardiovascular diseases in children is 5–16% globally [1]. Obstructive lesions of different segments of the right ventricular outflow tract (RVOT) are congenital heart defects, the treatment of which is possible only by surgical intervention. A modified Blalock–Taussig shunt (MBTS) is adopted as the first step in surgical treatment of RVOT. A shunt made of polytetrafluoroethylene (PTFE, Gore-Tex) is used to provide blood flow from the systemic circulation to the pulmonary circulation. In particular, the following pathologies are considered in the present study: pulmonary artery stenosis, pulmonary atresia, and Tetralogy of Fallot. However, MBTS operations cause a number of complications, such as excessive volumetric load, acute thrombosis, and low diastolic blood pressure leading to coronary insufficiency. Modern modeling techniques can be applied to predict and evaluate complications in the postsurgical period.

Computational modeling is used to study the local hemodynamics of MBTS surgery. The first such studies using numerical simulation were conducted in [2,3] using the computational fluid dynamics (CFD) method. The shunt size, its location, and its angles of proximal anastomoses for an idealized geometry were considered in these works. A similar study was [4]. CFD is a method determining the parameters of flow in a channel with solid walls. It allows for a detailed description of the local flow in a complex geometry. The hemodynamic effects of nonclosure of patent ductus arteriosus [5] on pulmonary blood flow were analyzed using the CFD method. The degree of shunt occlusion after MBTS surgery using CFD was determined by Arthurs et al. [6]. MBTS and right ventricle–pulmonary artery shunts were compared in [7,8]. Liu et al. conducted a series of studies [9,10,11,12] of MBTS shunts using real geometry to analyze the hemodynamic parameters of local blood flow, including the degree of shunt occlusion. In [10,13], the effect of MBTS location on blood flow distribution was investigated. Using the CFD method, the hemodynamics of the central shunt and modified Blalock–Taussig shunt were compared [14].

Vascular walls are flexible, which can affect the hemodynamics. The fluid–solid interaction (FSI) method is another method of computer modeling. This method, as opposed to the CFD method, takes into account the wall elasticity of the computational flow area. This numerical method is used to study the parameters of blood flow for different problems. Luo et al. [15] studied fluid flow in aortic and iliac arteries bifurcations with the use of FSI simulation. Stergiou et al. [16] investigated the occurrence and development of abdominal aortic aneurysm using the FSI method. Sousa et al. [17] analyzed the hemodynamics of the stenosis carotid bifurcation by FSI simulation. The deformability/elasticity of the vessel wall is an important factor in the study of blood flow. FSI simulation has not yet been used to study MBTS surgery. Therefore, this method was used in our study.

In this study, the shunt walls were modeled as an isotropic hyperelastic material, while the aortic walls were modeled as both anisotropic and isotropic elastic material. An experimental study of Gore-Tex shunts was performed to determine the parameters of the hyperelastic material of the shunt. FSI modeling of MBTS bypass surgery is an actual research method and has not been used before. The local distribution of velocities, pressures, wall shear stresses, and displacements of the aortic wall and shunt were analyzed in this study. The aim of the study was to show the effect of anisotropy on the main characteristics of local hemodynamics for the MBTS surgery. Results for the three methods for different shunt locations (central, right, and left) were obtained in this study.

## 2. Materials and Methods

Complex research, including experimental and computational studies, was carried out. The experimental part included tensile tests of artificial vascular grafts to determine mechanical properties and constitutive relation constants. FSI simulations of blood flow in the aorta after modified Blalock–Taussig shunt surgery were also performed. The results were analyzed for different shunt locations. The influence of the aortic mechanical properties on the local hemodynamics was studied.

### 2.1. Experimental Study on Mechanical Properties of Grafts

The study investigated the mechanical properties of Gore-Tex vascular grafts. These are most frequently used in surgical practice for shunt placement, including the modified Blalock–Taussig shunt operation. The Gore-Tex vascular graft is a tube made of a special material. It is a waterproof and vaporizable membrane. Constants of the strain energy density function for the hyperelastic material were obtained as a result of the experiments. The influence of the wall mechanical properties on the blood flow in an artificial vessel was evaluated considering the interaction between the vessel wall and the blood flow. The study employed the methods of computational fluid dynamics and mechanics. Calculation results were obtained for elastic and hyperelastic walls. The research considered local hemodynamics in the aorta with regard to anisotropy and hyperelastic properties; FSI modeling methods were applied. The results were analyzed to deduce the main factors influencing the local blood flow.

**Figure 1:**
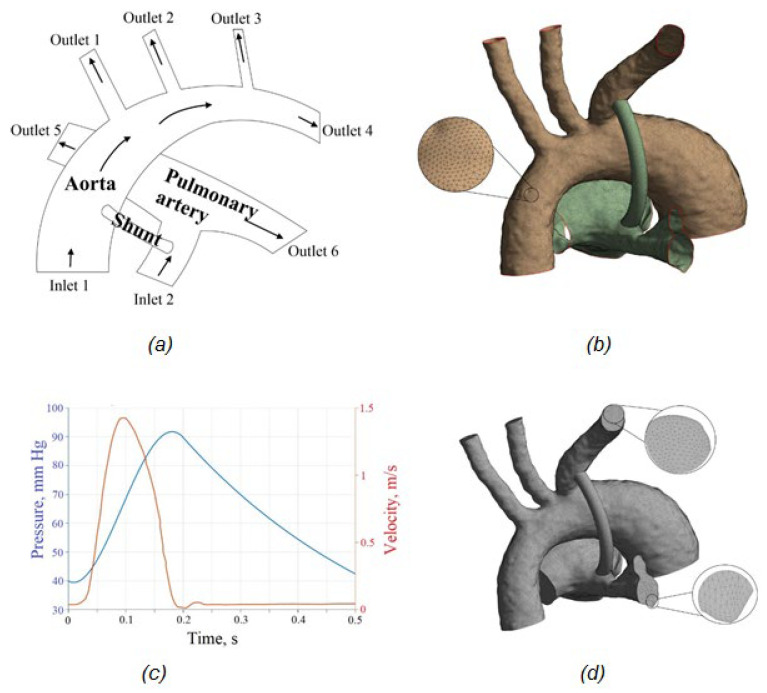
Meshes and boundary conditions of the aorta–shunt–pulmonary artery system: (**a**) boundary conditions, (**b**) solid mesh, (**c**) velocity and pressure profiles, (**d**) aorta fluid mesh model.

Gore-Tex shunts (W. L. Gore & Associates, Inc., Flagstaff, AZ, USA) of different sizes and thickness were used for tests (Figure 1). Currently, PTFE shunts are actively used in surgery. The test temperature was 37 °C. Experiments were performed under different loading to assess the effect of load rate on deformation.

Most human tissues behave nonlinearly under a load. Such materials are called hyperelastic. A strain energy density function is used to describe the behavior of hyperelastic materials under a load.

The materials are incompressible. The results of the experimental study can be described by the five-parameter Mooney–Rivlin model:

(1) $${W=c}_{10}(\bar{I_{1}}{-3)+c}_{01}(\bar{I_{2}}{-3)+c}_{20}{(\bar{I_{1}}-3)}^{2}+c_{11}(\bar{I_{1}}-3)(\bar{I_{2}}-3)+c_{02}{(\bar{I_{2}}-3)}^{2}+\frac{1}{D_{1}}{\left(J-1\right)}^{2}$$

and the three-parameter Yeoh model:

(2) $$W=\sum_{i=1}^{3}c_{i0}{\left(\bar{I_{1}}-3\right)}^{i},$$

where $c_{\mathrm{ij}}$ are material constants; $\bar{I_{1}}$, $\bar{I_{2}}$ are the first and the second invariant of the deviatoric strain tensors:

(3) $${\bar{I}}_{1}{=\lambda }_{1}^{2}+\lambda_{1}^{2}+\lambda_{1}^{2},$$

(4) $${\bar{I}}_{2}{=\lambda }_{1}\lambda_{2}+\lambda_{2}\lambda_{3}+\lambda_{1}\lambda_{3},$$

(5) $${\bar{I}}_{3}=\lambda_{1}^{2}\lambda_{1}^{2}\lambda_{1}^{2},$$

where $\lambda_{i}$ are principal stretches in Equations (3)–(5). The materials are incompressible; $\bar{I_{3}}=1$.

The constants in the strain energy density function (Equations (1)–(2)) were determined in the ANSYS Workbench software (Ansys Workbench 18, Ansys Inc., Canonsburg, PA, USA) using a curve fitting procedure based on the experimental tensile diagrams obtained for the sample. The five-parameter Mooney–Rivlin model (Equation (1), Equations (3)–(5)) and the three-parameter Yeoh model (Equations (2)–(5)) are used to describe the behavior of mechanical properties of grafts in this study.

### 2.2. Mechanical Properties of Aorta

#### 2.2.1. Ogden Model for Description of Isotropic Hyperelastic Behavior of Aorta

In the Ogden material model (Equation (6)), the strain energy density is expressed in terms of the principal stretches as:

(6) $${W(\lambda }_{1}{,\;\lambda }_{2}{,\;\lambda }_{3}\;)=\overset{N}{\underset{p=1}{\sum }}\frac{\mu_{p}}{\alpha_{p}}\left(\lambda_{1}^{\alpha_{p}}+\lambda_{2}^{\alpha_{p}}+\lambda_{3}^{\alpha_{p}}-3\right),$$

where N, $\mu_{p}$, and $\alpha_{p}$ are material constants. Under the assumption of incompressibility, one can rewrite this as

(7) $${W(\lambda }_{1}{,\;\lambda }_{2}\;)=\sum_{p=1}^{N}\frac{\mu_{p}}{\alpha_{p}}\left(\lambda_{1}^{\alpha_{p}}+\lambda_{2}^{\alpha_{p}}+\lambda_{1}^{-\alpha_{p}}\lambda_{2}^{-\alpha_{p}}-3\right).$$

In general, the shear modulus is calculated as follows:

(8) $$2\mu =\sum_{p=1}^{N}\mu_{p}\alpha_{p}.,$$

where N = 3; by fitting the material parameters, the material behavior of rubbers can be described very accurately. For particular values of material constants, the Ogden (Equation (7)) model will reduce to either the neo-Hookean solid (N = 1, α = 2) or the Mooney–Rivlin material (N = 2, α_1_ = 2, α_2_ = −2 with the constraint condition λ_1_ λ_2_λ_3_ = 1).

#### 2.2.2. Holzapfel–Gasser–Ogden Model for Description of Anisotropic Hyperelastic Behavior of Aorta

The simplified form of the strain energy potential is based on that proposed by Holzapfel, Gasser, and Ogden [18] for modeling arterial layers with distributed collagen fiber orientations:

(9) $${W=C}_{10}\left({\bar{I}}_{1}-3\right)+\frac{k_{1}}{2k_{2}}\overset{N}{\underset{\alpha =1}{\sum }}\left\{\exp\left[k_{2}{〈\bar{E}}_{\alpha }〉{}^{2}\right]-1\right\},$$

with

(10) $${\bar{E}}_{\alpha }=\kappa \left({\bar{I}}_{1}-3\right)+\left(1+3\kappa \right)\left({\bar{I}}_{4\left(\alpha \alpha \right)}-1\right),$$

where W is the strain energy per unit of reference volume; C_10_, κ, k_1_, and k_2_ are temperature-dependent material parameters; N is the number of families of fibers (N ≤ 3); ${\bar{I}}_{1}$ is the first deviatoric strain invariant; and ${\bar{I}}_{4\left(\alpha \alpha \right)}$ are pseudo-invariants of $\bar{C}$ and ${\bar{E}}_{\alpha }$.

The model (Equations (9)–(10)) assumes that the directions of the collagen fibers within each family are dispersed (with rotational symmetry) about a mean preferred direction. The parameter κ (0≤κ≤1/3) describes the level of dispersion in the fiber directions. If ρ(Θ) is the orientation density function that characterizes the distribution (it represents the normalized number of fibers with orientations in the range [Θ,Θ+dΘ] with respect to the mean direction), the parameter κ is defined as

(11) $$\kappa =\frac{1}{4}\int_{0}^{\pi }\rho \left(\Theta \right)\sin^{3}\Theta d\Theta .$$

It is also assumed that all families of fibers have the same mechanical properties and the same dispersion (Equation (11)). When κ = 0, the fibers are perfectly aligned (no dispersion). When κ = 1/3, the fibers are randomly distributed and the material becomes isotropic; this corresponds to a spherical orientation density function.

In this study, isotropic (Equations (7)–(8)) and anisotropic (Equations (9)–(11)) Holzapfel–Gasser–Ogden models are applied to describe the hyperelastic properties of the aorta.

### 2.3. FSI Simulations of Blood Flow in the Aorta–Pulmonary Artery–Shunt System

#### 2.3.1. Problem Formulation

A concomitant pathology of congenital heart disease in children is impaired pulmonary circulation. This leads to abnormal lung growth and insufficient oxygenation. The use of a modified Blalock–Taussig shunt (MBTS) in such cases is one of the most common methods for eliminating pathology. Biomechanical modeling methods are used to objectify the choice of shunt parameters. An individualized three-dimensional model of the aorta–pulmonary artery–shunt system based on CT images (computed tomography) with contrast was built to analyze the local hemodynamics.

The study’s protocol was approved by the Ethics Committee of the Perm Federal Center of Cardiovascular Surgery (Protocol No. 12 on 25 October 2021). Informed consent was obtained from parents of patients involved in the study.

Three-dimensional (3D) anatomical data were obtained via a 64-channel, dual-source multidetector-row CT scanner (Siemens Somatom Definition AS, Forchheim, Germany) with a 0.6-mm slice thickness and 0.6-mm slice interval, a 0.5 s rotation time, and a pitch of 0.25 (Gantry opening is 70 cm; the number of reconstructed slices is 67). The tube current was adjusted according to the body weight. Before CT examination, all patients were sedated. Intravenous propofol was given by anesthesiologist at a dose of 1–2 mg/kg body weight for induction. In some cases, the dose was increased to maintain sedation. Anatomical coverage extended from above the thoracic inlet to below the level of the L2 vertebra, including the origin of the celiac trunk. For vascular opacification, a non-ionic low-osmolar contrast agent containing 350 mg/mL was injected through the peripheral vein (the right ulnar vein if it was accessible). Contrast was administered with a mechanical injector at a dose of 2 mL/kg body weight. A flow rate of 1–2 mL/s was used, depending on the size and location of the venous access, as well as the size of the cannula used. Postprocessing was carried out on a dedicated workstation Singo via (Siemens Healthcare GmbH, Erlangen, Germany). Image reconstruction was performed in 3D volume rendering (VRT) maximum intensity projection (MIP) of multiplanar reconstruction (MPR) in coronary, sagittal oblique views.

Three variants of a modified Blalock–Taussig shunt were considered: the central, connecting the aorta with the pulmonary artery trunk; the right, connecting the left subclavian artery and the right pulmonary artery; and the left, connecting the brachiocephalic trunk and the left pulmonary artery.

The results obtained for two modifications of the model were compared: the first model the so-called “simplified” model accounting for the aortic wall’s isotropy and the shunt elastic properties; the second model accounting the aortic wall’s anisotropy and the shunt hyperelasticity. The calculations were performed in Ansys Workbench software (Ansys Workbench 18, Ansys Inc., Canonsburg, PA, USA). The two-way fluid–solid interaction problem of blood flow in the aorta–pulmonary artery system in children was solved.

#### 2.3.2. Mathematical Problem Statement

The mass and momentum conservation equations (Equations (12)–(13)) for an incompressible fluid (Equation (14)) can be expressed as

(12) $$\rho_{f}\left(\frac{\partial u}{\partial t}+\left(u\cdot \nabla \right)u\right)=\nabla \cdot \sigma_{}$$

(13) ∇·u=0,

(14) σ=−pI+τ

(15) $$\tau =\eta \left(\dot{\gamma }\right)D,$$

where ρ_f_ is the fluid density, p is the pressure, u is the fluid velocity vector, and u_g_ is the moving coordinate velocity. In the arbitrary Lagrangian–Eulerian (ALE) formulation, (u−u_g_) is the relative velocity of the fluid with respect to the moving coordinate velocity. Here, τ is the deviatoric shear stress tensor (Equation (15)). This tensor is related to the velocity through the strain rate tensor; in Cartesian coordinates it can be represented as follows:

(16) $$D=\frac{1}{2}(+\text{∇}u^{T}).$$

The momentum conservation equation for the solid body can be written as follows:

(17) $$\nabla \cdot \sigma_{s}=\rho_{s}\ddot{u_{s}},$$

where $\rho_{s}$, $\sigma_{s}$, and $\ddot{u_{s}}$ are the density, stress tensor, and local acceleration of the solid, respectively.

It is known that blood vessels can be described as hyperelastic materials [18,19,20]. Because of a similar anatomical composition, the bile ducts can also be considered hyperelastic materials. For hyperelastic materials, the stress–strain relationship is written as follows:

(18) $$\sigma_{s}=\frac{\partial W}{\partial \varepsilon },$$

where ε is the strain tensor and W is the strain energy density function. The Mooney–Rivlin hyperelastic potential is shown in Equation (1).

The mathematical statement of blood flow in the aorta–shunt–pulmonary artery system is governed by Equations (12)–(18)).

The FSI interface should satisfy the following conditions:

(19) $$x_{g}=x_{s}$$

(20) $$u_{g}=u_{s}$$

(21) $$\sigma_{g}{\hat{n}}_{g}=\sigma_{s}{\hat{n}}_{s}.$$

The displacements of the fluid and solid domain should be compatible, as in Equation (19). The tractions at this boundary must be at equilibrium (Equation (21)). The no-slip condition for the fluid should satisfy Equation (20). In the above conditions, Equations (19)–(21) give the displacement, stress tensor, and boundary normal, respectively. The subscripts f and s indicate fluid and solid parts, respectively. Blood is assumed to be a Newtonian fluid. The blood density is equal to ρ=1060 kg/m^3^; the dynamic viscosity is constant and equal to μ=0.0035 Pa·s. The velocity profile during the systolic and diastolic phases of the left ventricle was applied at the aortic root inlet (Figure 1). The left ventricular systole period is t = 0.22 s. The period of ventricular diastole is t = 0.28 s. The total cardiac cycle is t = 0.5 s. The peak velocity is 1.4 m/s. A time-dependent pressure profile was used as the boundary conditions at the aortic outlets. Constant pressure of P = 20 mm Hg was applied at the pulmonary artery outlets.

#### 2.3.3. Mesh and Convergence

The computational mesh of the fluid domain was generated using the Body Sizing and Inflation tools, respectively. Body Sizing allows one to set the mesh item type and size. The Inflation tool allows one to thicken the mesh in the near-wall regions to further reveal the near-wall effects (Figure 1a). The computational mesh for the solid domain was selected based on the study of the mesh convergence of the results.

**Figure 2:**
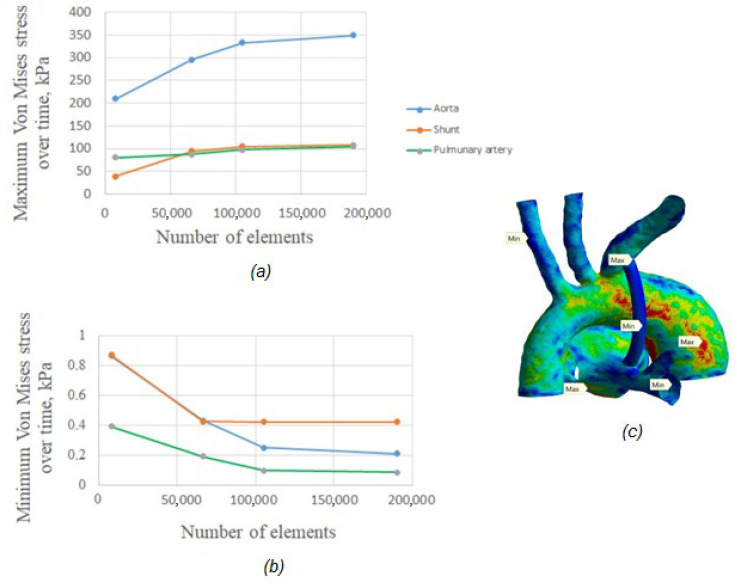
Maximum stress variations with respect to the number of mesh elements for solid domain: (**a**) mesh dependency tests for maximum von Mises stress, (**b**) mesh dependency tests for minimum von Mises stress, (**c**) maximum and minimum values for aorta, shunt, and pulmonary artery.

Five different element sizes were selected to analyze the sensitivity to the grid density (Table 1). The element types used in all grids were hexahedral and tetrahedral. An analysis of the sensitivity to the mesh density was carried out based on the achievement of a relative difference $\varepsilon_{P}^{\min}\;$ = 0.21%, $\varepsilon_{V}^{\min}\;$ = 0.84% of the variation of the maximum values of pressure and velocity in the aorta–shunt–pulmonary artery system. Figure 2 shows a convergence plot for von Mises stress. The results of the study showed that the values of the maximum stress values for a coarse mesh differ significantly from a thickened mesh. Thus, for subsequent calculations, a denser mesh with a side size of a triangular finite element h = 0.2 mm was used.

## 3. Results

### 3.1. Results of the Experimental Study

**Figure 3:**
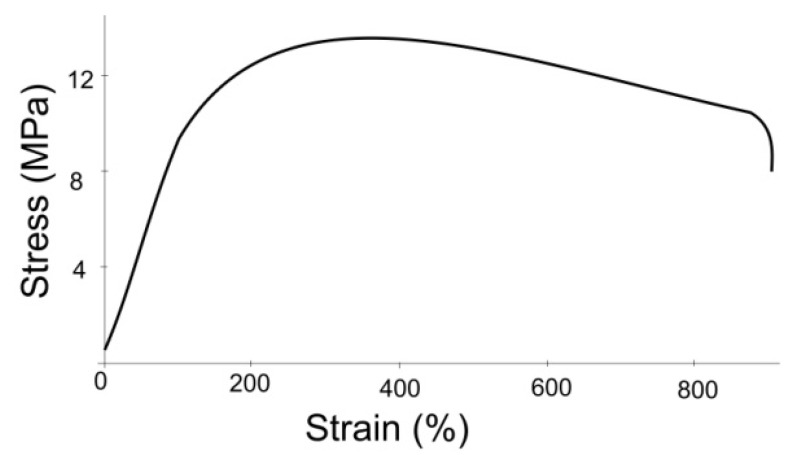
Stress–strain diagram by specimen rupture test.

Tensile and rupture tests were carried out. Various factors were analyzed, including the loading rate and geometric dimensions of the specimen. As a result of the experiment, the modulus of elasticity was determined for different specimens (Table 2). The elasticity modulus strongly depends on the diameter and thickness of the shunt: for a thickness over 0.5 mm, its value increases several times, and for a thickness less than 0.35 mm, it decreases strongly. The stress–strain curve for a shunt with a diameter of 4.5 mm, wall thickness of 0.35 mm, and length of 20 mm by a rupture test is shown in the Figure 3. The load rate was 30 mm/min and the preload was 0.5 MPa.

**Figure 4:**
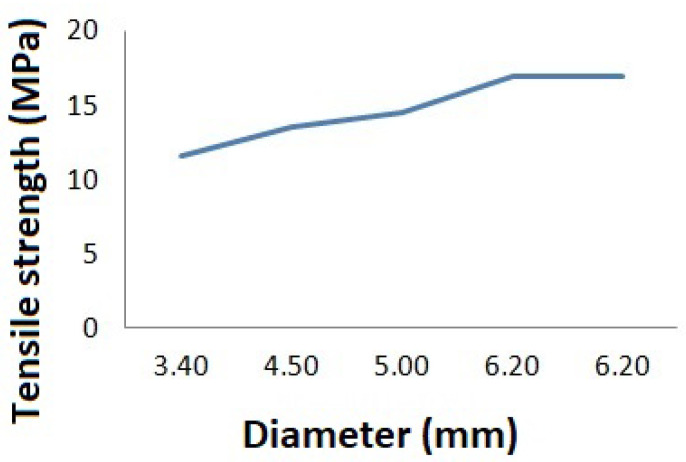
Plot of change in ultimate strength with specimen diameter.

**Figure 5:**
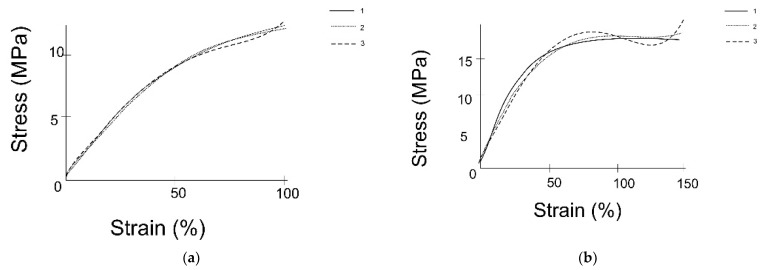
Stress–strain diagrams: (**a**) specimen 1, (**b**) specimen 2. The solid line (1) is the experimental curve, the dashed line (2) is the five-parameter Mooney–Rivlin strain energy density function, and the dotted line (3) is the three-parameter Yeoh strain energy density function.

The tensile ultimate strength σ_Y_ was also determined (Table 3); its value increases as the specimen diameter increases (Figure 4). The influence of loading rate on tensile strength σ_Y_ determination was analyzed. The tensile strength value remained practically unchanged when the load rate application changed from 50 to 250 mm/min. The shape of the tensile test curve for all specimens was the same. Stress–strain relationships were obtained as a result of tensile tests for two specimens (specimen no. 1, diameter of 5 mm, thickness of 0.5 mm, length of 20 mm; specimen no. 2, diameter of 3 mm, thickness of 0.35 mm, length of 20 mm). The constants for the strain density function were determined from the obtained dependences (Table 4) and stress–strain dependences were plotted (Figure 5).

### 3.2. Results of FSI Simulation of the Blood Flow

As a result of solving the problem, the distributions of hemodynamic parameters were obtained from three patients, including blood flow velocity, pressure, wall shear stress, time-averaged wall shear stress, and other parameters. The mechanical properties of the aorta–pulmonary artery–shunt system are shown in Table 5 in the considered computational domain. The most important results from the hemodynamic point of view were obtained at t = 0.09 s, corresponding to the maximum blood flow velocity. Similar results obtained for simple geometry (straight vessel) are presented in Supplementary Materials.

#### 3.2.1. Velocity Distribution

**Figure 6:**
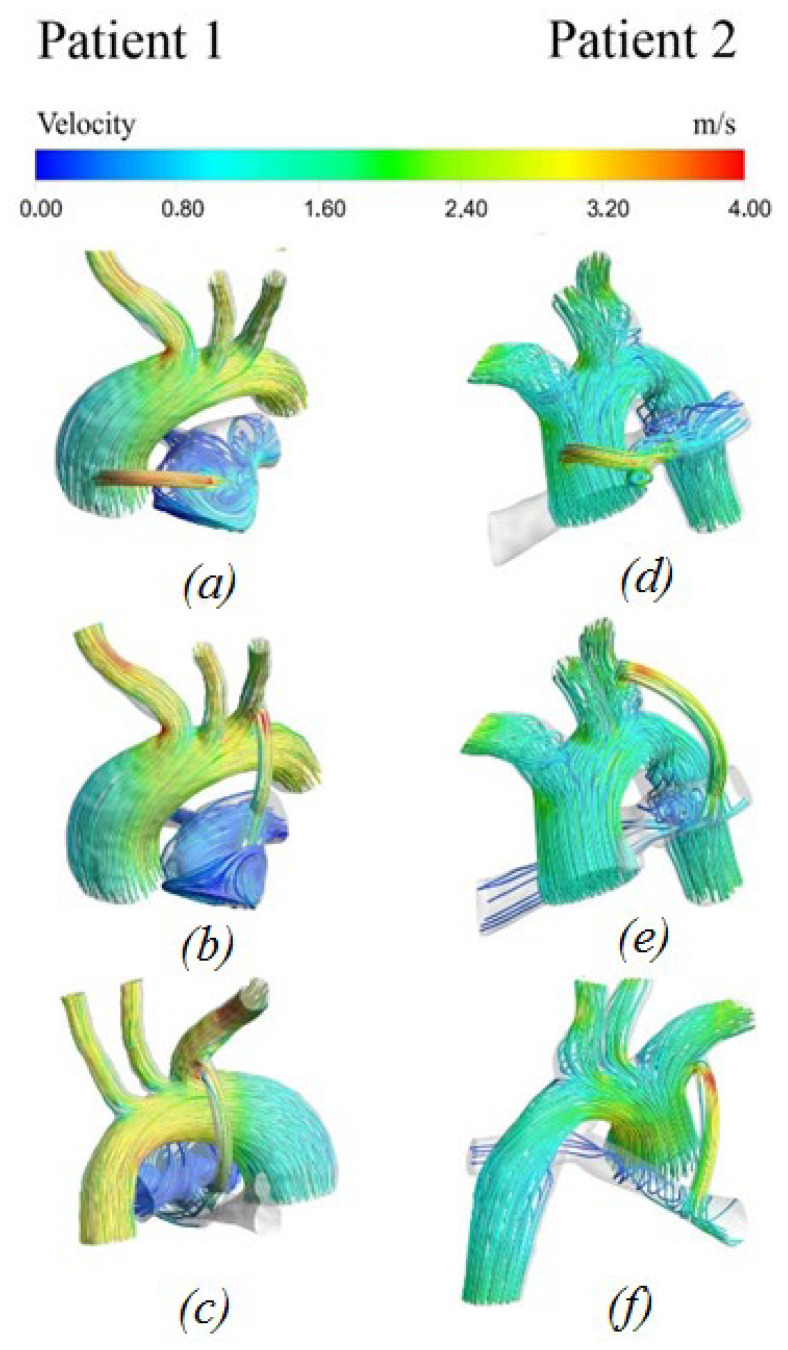
Velocity distribution with anisotropic properties of the aorta and hyperelastic properties of the shunt: (**a**,**d**) central shunt; (**b**,**e**) right shunt; (**c**,**f**) left shunt.

Figure 6 shows the distribution of blood flow velocity characteristics. In the area of the aorta, the blood flow has a uniform distribution pattern. There is a local increase in the rate of blood flow in the region of the descending aorta and bifurcations. As one moves away from the descending part of the aorta, the blood flow velocity is equalized. The reverse situation is true in the pulmonary artery. In the pulmonary artery, there is mainly a vortex flow of blood in all patients. At the peak moment of systole, the maximum values of blood flow are observed in the area of the shunt.

#### 3.2.2. Pressure distribution

**Figure 7:**
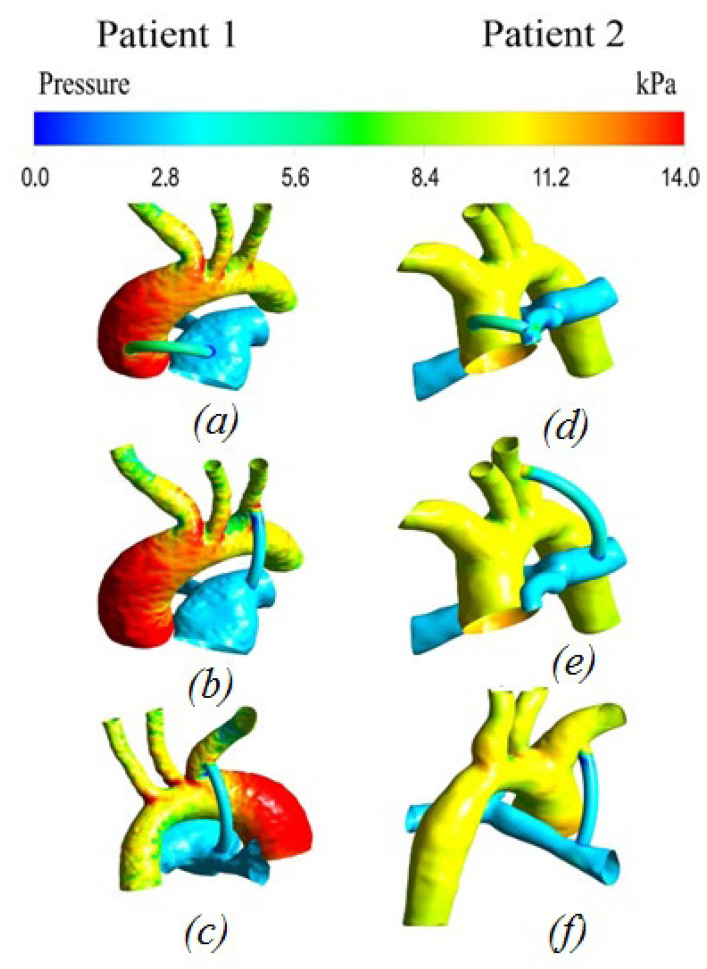
Pressure distribution with anisotropic properties of the aorta and hyperelastic properties of the shunt: (**a**,**d**) central shunt; (**b**,**e**) right shunt; (**c**,**f**) left shunt.

Figure 7 shows the pressure distribution at the peak moment of systole. The distribution of pressure along the walls of the aorta and pulmonary artery is uneven. The highest values are concentrated on the walls of the ascending part of the aorta and its branches (left subclavian artery, left common artery, and brachiocephalic trunk), while the lowest values are observed on the walls of the pulmonary artery and shunt.

In the shunt zone, the maximum values are concentrated in the area of the junction with the aorta, then the pressures are distributed evenly up to the pulmonary artery.

#### 3.2.3. Wall Shear Stress

The distribution of shear stresses is important in the study of systemic blood flow. In the literature, particular importance is given to the distribution of the shear-wall shear stresses. Most authors associate hypoplasia of the intima of the vascular bed with high shear stress [21].

The wall shear stress indicates two problems: lipids remain on the vessel wall at low values, and they damage the vessel wall at high values, which also increases the ability of lipids to linger on the damaged intima**.**

**Figure 8:**
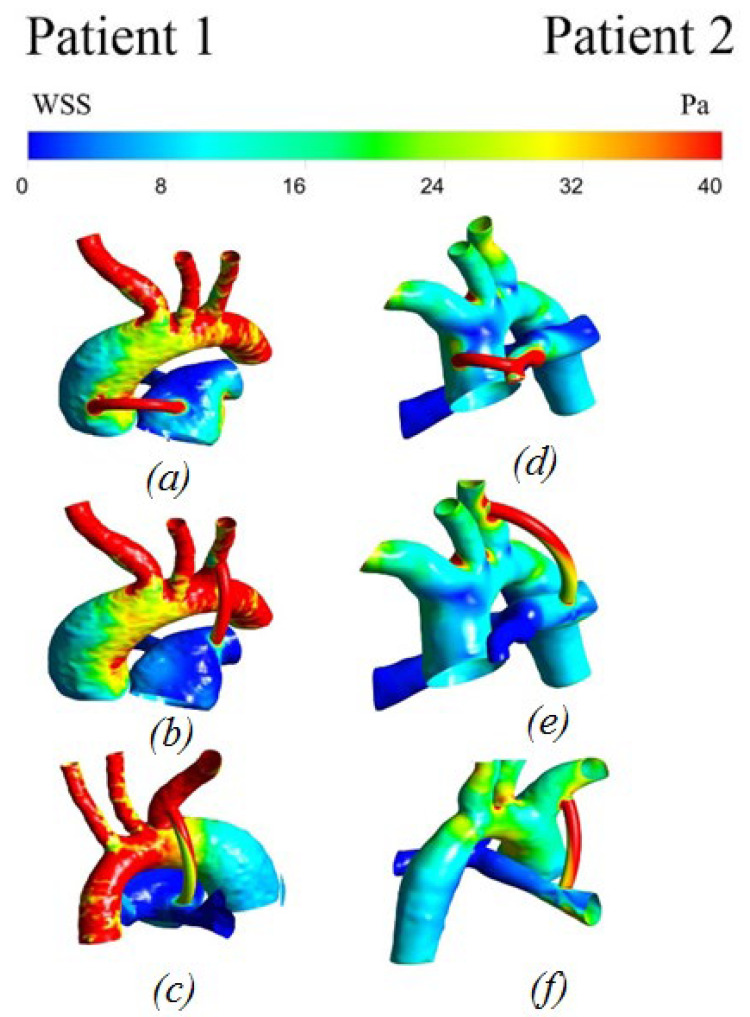
Distribution of wall shear stress with anisotropic properties of the aorta and hyperelastic properties of the shunt: (**a**,**d**) central shunt; (**b**,**e**) right shunt; (**c**,**f**) left shunt.

Figure 8 shows the distribution of wall shear stress. The highest values are localized in the area of the shunt, which can lead to its thrombosis. Additionally, large values of parietal shear stresses are concentrated in the pulmonary artery in the vortex, with stagnant blood flow on the branches of the aorta (left subclavian artery, left common artery, and brachiocephalic trunk). The minimum values are observed in the areas of the descending part of the aorta and the beginning of the right and left pulmonary arteries.

#### 3.2.4. Distribution of Time-Averaged Shear Stress

**Figure 9:**
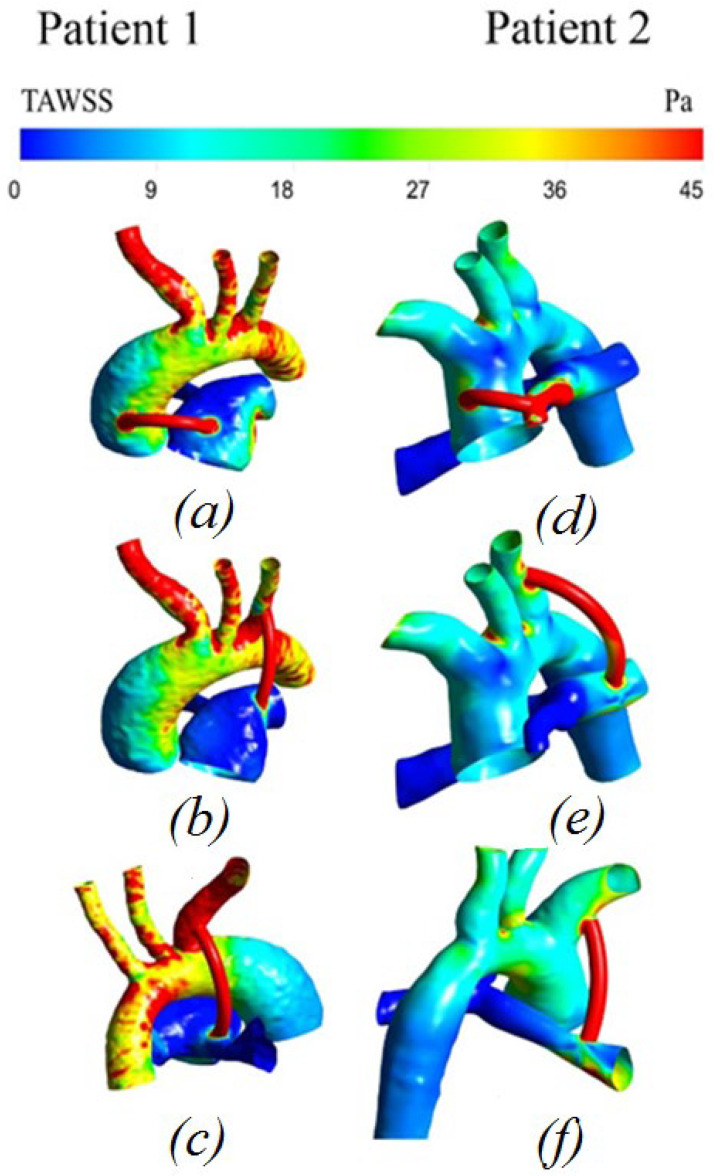
Distribution of time-averaged wall shear stress with anisotropic properties of the aorta and hyperelastic properties of the shunt: (**a**,**d**) central shunt; (**b**,**e**) right shunt; (**c**,**f**) left shunt.

Figure 9 shows the time-averaged shear stress. The values of the shear stress at the peak moment of systole are highest in the shunt area, causing shunt thrombosis. Additionally, large values are located in the area of vortex movement of blood in the underlying region of the pulmonary artery, as well as in local areas of the branches of the aorta, due to the special geometric characteristics of each geometry of patients.

#### 3.2.5. Displacement Distribution

**Figure 10:**
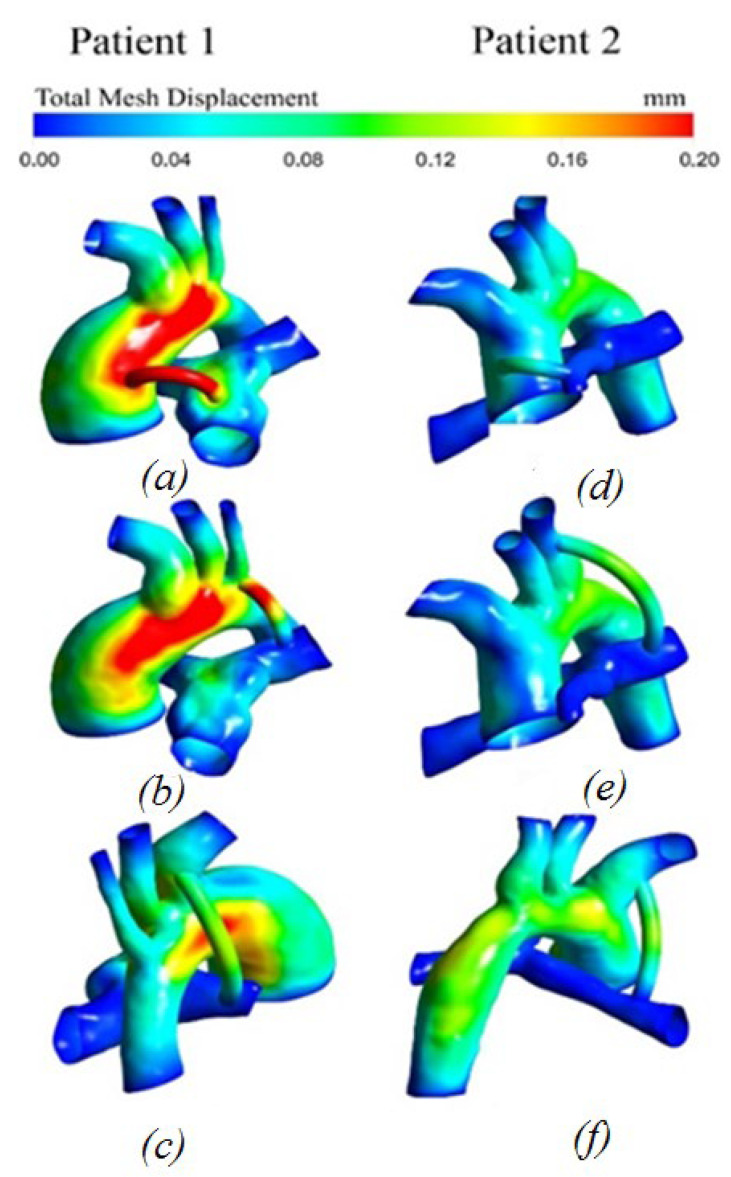
Distribution of displacements with anisotropic properties of the aorta and hyperelastic properties of the shunt: (**a**,**d**) central shunt; (**b**,**e**) right shunt; (**c**,**f**) left shunt.

Figure 10 shows the distribution of displacements occurring in the system. The displacement values at the peak moment of systole are highest in the area of the shunt and the lateral part of the aorta with the central and right location of the shunt. With the left-sided shunt position, the maximum values are distributed only along the lateral part of the aorta.

#### 3.2.6. Von Mises Stress Distribution

**Figure 11:**
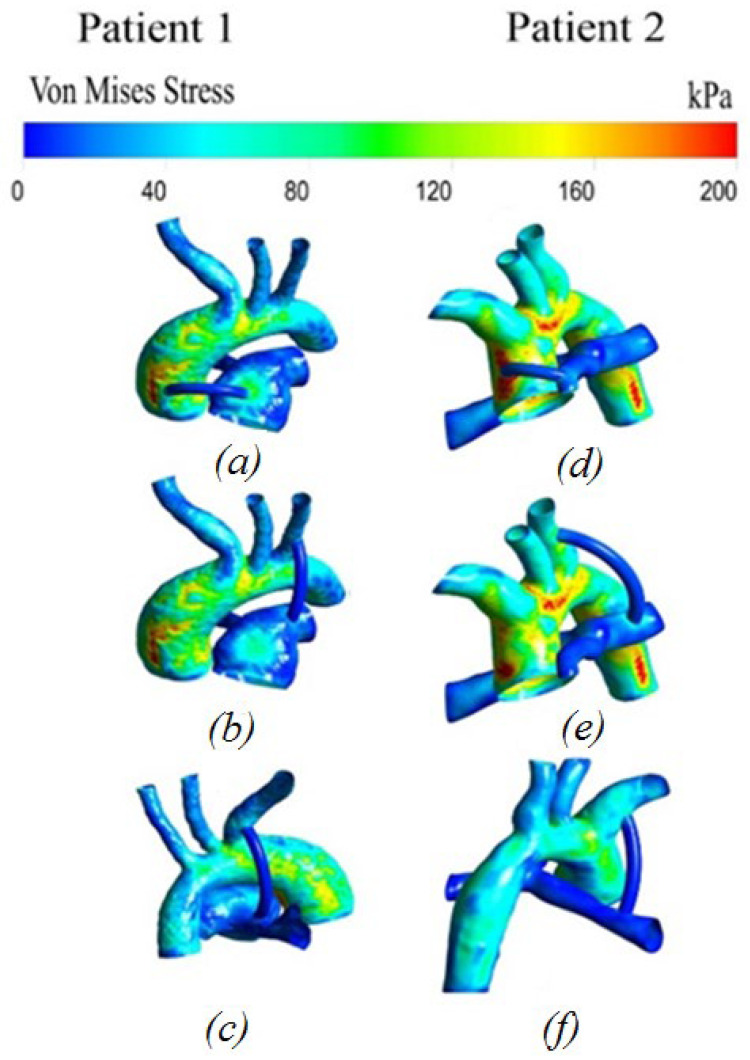
Distribution of stresses in the case of anisotropic properties of the aorta and hyperelastic properties of the shunt: (**a**,**d**) central shunt; (**b**,**e**) right shunt; (**c**,**f**) left shunt.

Figure 11 shows the distribution of stresses arising in the system. The stress values at the peak moment of systole are highest in the areas of blood flow separation and have a non-uniform distribution pattern. Additionally, high values are located in local areas of the aorta, characterized by the unevenness of the walls of the system.

## 4. Discussion

### 4.1. Difference between Isotropic and Anisotropic Models

**Figure 12:**
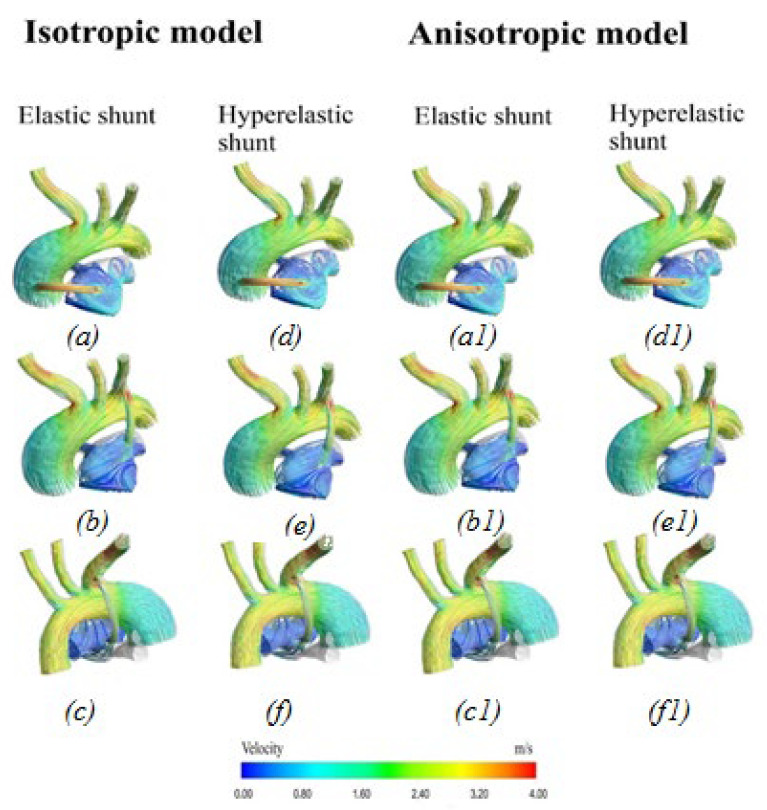
Velocity distribution: (**a**,**d**,**a1**,**d1**) central shunt; (**b**,**e**,**b1**,**e1**) right shunt; (**c**,**f**,**c1**,**f1**) left shunt.

**Figure 13:**
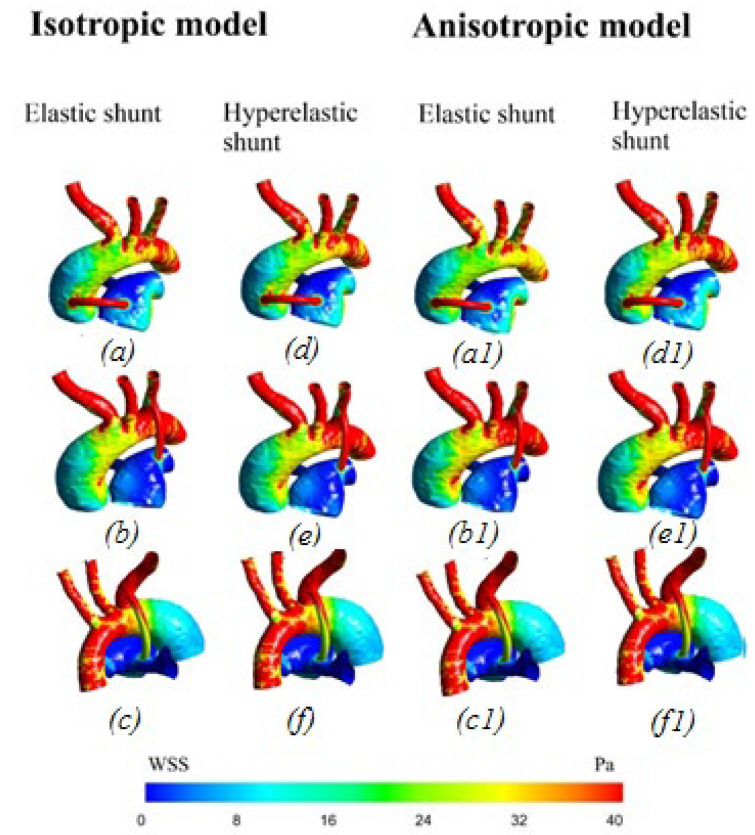
Wall shear stress distribution: (**a**,**d**,**a1**,**d1**) central shunt; (**b**,**e**,**b1**,**e1**) right shunt; (**c**,**f**,**c1**,**f1**) left shunt.

The distribution of hemodynamic parameters in the anisotropic and isotropic models of materials in patients has the same distribution pattern throughout the system. The dynamics of blood flow is identical in all patients. The numerical values are also the same (Figure 12 and Figure 13). The differences between the anisotropic and isotropic properties of the aorta and the pulmonary artery are noticeable only when analyzing the stress–strain state, i.e., in displacements and stresses arising in the aorta–shunt–pulmonary artery system.

**Figure 14:**
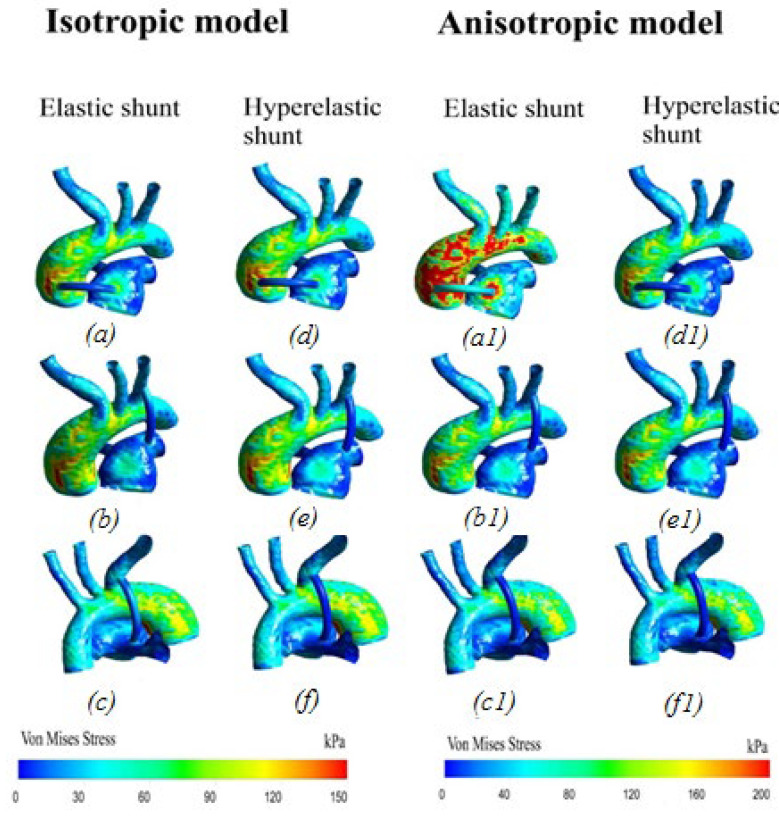
Von Mises stress distribution: (**a**,**d**,**a1**,**d1**) central shunt; (**b**,**e**,**b1**,**e1**) right shunt; (**c**,**f**,**c1**,**f1**) left shunt.

The opposite situation is seen with von Mises stress distribution (Figure 14). There is also a similar pattern of stress distribution throughout the system, with the exception of the central location of the shunt, where, according to the anisotropic model, increased stress values are mostly observed in the aorta. In addition, the anisotropic model of the material shows higher stress values than the isotropic model of the aortic material. The maximum stress on the wall of the anisotropic aorta is about 200 kPa; the maximum stress on the wall of the isotropic aorta is about 150 kPa.

Along with the hemodynamic parameters, the parameters of the stress–strain state, such as displacements and von Mises stress, also affect the success of a surgical intervention [22].

It was shown that the anisotropic model of the aortic material shows higher stress values at the peak moment of systole, which in turn may be a key factor in determining the strength characteristics of the aorta and pulmonary artery, all other things being equal. Additionally, this mechanical parameter is important when installing a central shunt, since it is in the area of the central anastomosis that an increase in stresses on the aortic wall is observed.

Displacement distribution is also important. According to the computations, the anisotropic model shows smaller values of the displacements of both the aorta and the shunt, which in turn may affect the success of preoperative prediction. Thus, it can be concluded that the anisotropic properties of the aorta play an important role in preoperative modeling.

**Figure 15:**
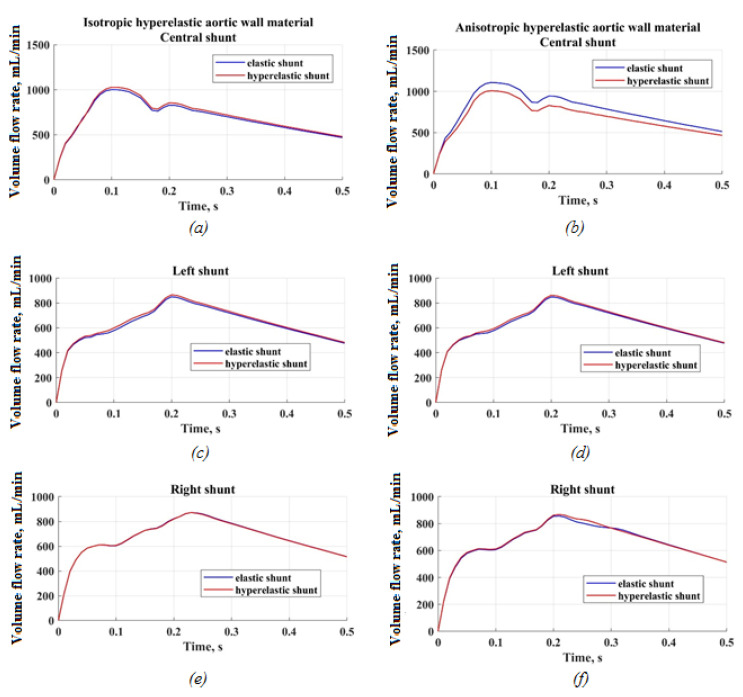
Flow rate through the shunt: (**a**) central shunt case (isotropic aortic wall), (**b**) central shunt case (anisotropic aortic wall), (**c**) left shunt case (isotropic aortic wall), (**d**) left shunt case (anisotropic aortic wall), (**e**) right shunt case (isotropic aortic wall), (**f**) right shunt case (anisotropic aortic wall).

The time dependences of the volumetric flow rate of blood flow inside the shunt show that, for all locations of the shunt and taking into account the hyperelasticity of the shunt, the results are almost identical (Figure 15). However, in the case of the central position of the shunt, when the aortic walls were considered anisotropic material, the volumetric flow of blood within the shunt was different. The maximum deviation of this value was 12%, at 0.2 s of the cardiac cycle. For the elastic shunt, the volumetric flow rate throughout almost the entire time exceeded the analogous value for the hyperelastic shunt. This difference is essential for the further correct formation of pulmonary blood flow.

Based on the same dependences of volumetric blood flow on time for the aortic wall, and taking into account its anisotropy, it can be concluded that this is reflected only in the case of a central shunt, when the shunt is an elastic material. Susequently, there is an excess of volumetric flow in 12% of cases with a hyperelastic shunt. Thus, taking into account the hyperelasticity of the shunts allows for obtaining more realistic results, reducing the possible negative consequences of operations.

Hemodynamic parameters are very important for the assessment of shunting [23]. To evaluate the effectiveness of shunting, it is worth considering indicators that can describe the probable risk of shunt thrombosis. Such indicators are wall shear stress and time-averaged wall shear stress.

As a result of the analysis of the distribution of indicators, high values of wall shear stress were revealed, and, consequently, time-averaged wall shear stress in the anastomotic region in all models. This, in turn, may indicate the risk of developing thrombosis [24,25,26]. It is also supported by the clinical and literature data [27,28,29].

### 4.2. Concluding Remarks

The obtained results of the distribution of von Mises stress show a doubling in this value for a central shunt, with an elastic material of the shunt in contrast to the hyperelastic material on the wall of the shunt itself. In addition, such a location is characterized by an increase in the stress values on the aortic wall and at the site of the shunt insertion into the pulmonary artery by 40–80 kPa. Taking into account the hyperelasticity of the shunt makes it possible to take into account some of its “damping” properties.

The values of shear stress on the wall for the central shunt also differ depending on the hyperelasticity of the shunt. Moreover, this is reflected in the descending aorta, where the difference is about 20%. It can be concluded that taking into account the hyperelasticity of the shunt material plays a particularly important role when the shunt is in the central position. In this case, the values of many hemodynamic characteristics change, which will further affect the development of shunt thrombosis and the distribution of pulmonary blood flow.

### 4.3. Limitations

We recognize several limitations of our study. Three variants of a modified Blalock–Taussig shunt were considered, central, right, and left, but these models are not always possible to realize. There are other organs nearby that can make it impossible to implement certain shunt locations.

The study does not present a retrospective clinical analysis of the MBTS operation for patients, which would allow us to objectify, to some extent, the calculation results obtained.

CT scans from only three patients were used for modeling in our study, which is a very small sample size. In future, with an increase in the number of subjects, these results can be used to guide clinical practice; this can be considered a pilot study towards this goal.

### 4.4. Possible Future Clinical Application

The development of non-invasive diagnostic and numerical methods in the contemporary surgery allows the estimation of the biomechanical processes in the human body. This circumstance increases the possibility of their use to improve existing and developing new personalized methods for diagnosing and predicting treatment. In particular, there is a growing need for the applications in the cardiovascular pediatric surgery.

Congenital heart disease is a general term for a range of birth defects that affect the normal way the heart works. The modified Blalock–Taussig shunt is commonly performed as early palliation in cyanotic congenital heart disease. One of the reasons is the use of subjective experience and the lack of individualized biomechanical models for the analysis of surgical interventions.

To predict and prevent postoperative complications, it is necessary to formulate and introduce new technological approaches, which, in particular, may consist in creating a software product (decision-making system in surgical interventions for gallstone disease and its complications). A proposed model of the blood flow in the system aorta–hunt–pulmonary artery makes it possible to assess hemodynamics in normal and pathological conditions, as well as to carry out a numerical assessment of modified Blalock–Taussig shunt to predict and prevent complications. The decision-making software based on such a biomechanical model will be able to evaluate the shunt position for the current patient, predict possible thrombosis risk, and evaluate mean flow rate after palliation surgery. Therefore, using the results of this paper, the surgeon can evaluate the circumstances of the operation for each patient before operation and evaluate the results of post-operative blood flow features.

## 5. Conclusions

There is no obviously ideal type and location for shunt insertion. Many studies have attempted to find a universal way to guide such a choice, in particular, using mathematical models. Our study offers a new step in the modeling of MBTS operations. In contrast to the well-known and more commonly used CFD method, we have proposed the FSI method, which takes into account not only the elasticity of the vessel wall but also its anisotropy. Changes in a number of the main hemodynamic parameters of local blood flow have been established depending on the accounting of elasticity properties. We consider this important for further research in modeling MBTS operations.

Modeling is an activity aimed at understanding and quantifying an event, object, or function. In state-of-the-art science, numerical modeling is often used to study a particular process. In our study, we also used already known tools (FSI) to simulate a specific event (MBTS). The use of the FSI method for MBTS operations has not been used before, so we called it a “new step”.

We have carried out complicated FSI modeling for MBTS operations, taking into account the anisotropic properties of vessel materials. No one has done this before. We have established the importance of taking anisotropy into account when modeling central bypass surgery. Therefore, we consider this a “new step” of modeling in MBTS operations.

A comparison between the effect of isotropic and anisotropic aorta material properties was performed. It was shown that the anisotropic model of the aortic material showed higher stress values at the peak moment of systole, which may be a key factor in determining the strength characteristics of the aorta and pulmonary artery. Additionally, this mechanical parameter is important when installing a central shunt, since it is in the area of the central anastomosis that an increase in stresses on the aortic wall is observed. According to computations, the anisotropic model shows smaller values of the displacements of both the aorta and the shunt, which in turn may affect the success of preoperative prediction. Thus, it can be concluded that the anisotropic properties of the aorta play an important role in preoperative modeling.

## Figures and Tables

**Table 1:** Parameters of samples used in the study.

No.	Body Sizing, mm	Inflation			Number of Elements	Maximum Pressure, Pa	Maximum Velocity, m/s
		Transition Ratio	Maximum Layers	Growth Ratio			
1	0.95	0.5	3	1.2	80,353	17,634	3.85
2	0.8	0.4	5	1.4	159,379	17,952	4.38
3	0.63	0.3	7	1.3	349,926	17,892	4.47
4	0.5	0.35	8	1.6	709,578	18,470	4.71
5	0.38	0.32	10	1.3	1,544,745	18,509	4.75

**Table 2:** Parameters of samples used in the study.

Sample Number	E (MPa)	Diameter, d (mm)	Wall Thickness (mm)
1	7.41	4.32	0.34
2	9.8	3.4	0.4
3	10.3	4.5	0.35
4	11.1	5.5	0.48
5	43.5	5	0.53

**Table 3:** Mechanical properties of samples.

Sample Number	σ_Y_ (MPa)	Diameter, d (mm)	Wall Thickness, (mm)	Loading Rate, (mm/min)
1	11.6	3.4	0.4	30
2	13.6	4.5	0.35	30
3	14.5	5	0.53	30
4	17.0	6.2	0.85	50
5	16.9	6.2	0.85	250

**Table 4:** Values of hyperelastic models for two samples.

Strain Density Function	Constants, Specimen No. 1 (MPa)	Constants, Specimen No. 2 (MPa)
The five-parameter Mooney–Rivlin model	$C_{10}\;=\;-1.64$, $C_{01\;}=\;2.59$, $C_{20}\;=\;4.46\times 10^{-7}$, $C_{11}\;=\;-2.39\times 10^{-4}$, $C_{02}\;=\;0.44$	$C_{10\;}=\;-2.2$, $C_{01}\;=\;3.26$, $C_{20}\;=\;3.86$, $C_{11}\;=\;-8.6\times 10^{-4}$, $C_{02}\;=\;0.62$
The three-parameter Yeoh model	$C_{10}\;=\;0.11$, $C_{20}\;=\;-4.96\times 10^{-6}$ $C_{30\;}=\;1.67\times 10^{-10}$	$C_{10}\;=\;0.20$ $C_{20}\;=\;-6.73\times 10^{-6}$ $C_{30\;}=\;1.16\times 10^{-10}$

**Table 5:** Mechanical parameters for aorta and shunt used in the study.

The Aorta		The Shunt	
Isotropic Hyperelastic Material	Anisotropic Hyperelastic Material)	Isotropic Elastic Material	Isotropic Hyperelastic Material
Ogden model: $\mu_{1}\;=\;1.274$ MPa $\mu_{2}\;=\;-1.211$ MPa $\alpha_{1}\;=\;24.074$ $\alpha_{2}\;=\;24.073$	Holzapfel–Gasser–Ogden model: $\mu_{1}\;=\;2.363$ MPa $\mu_{2}=0.839$ MPa $\alpha_{1}\;=\;0.6$ d = 0.001 MPa^−1^	E = 10.3 MPa μ = 0.49	Experimental data (Table 3)

## Data Availability

Not applicable.

## Supplementary Materials

The following supporting information can be downloaded at: https://www.mdpi.com/article/10.3390/ma15082719/s1. Table S1. Parameters of computational mesh., Table S2. Mechanical parameters for aorta and shunt used in the study., Figure S1. Meshes and boundary conditions of the "straight vessel": upper–boundary conditions and fluid mesh, lower–solid mesh., Figure S2. Stress distribution at time t = 0.09 s: (a) isotropic properties of the vessel, (b) anisotropic properties of the vessel., Figure S3. Displacements distribution at time t = 0.09 s: (a) isotropic properties of the vessel, (b) anisotropic properties of the vessel., Figure S4. Comparison of parameters: (a) maximum values of displacements, (b) maximum Von Mises stress values, (c) maximum wall shear stress values.
