# Supplementary material for: The Effects of the Mechanical Properties of Vascular Grafts and an Anisotropic Hyperelastic Aortic Model on Local Hemodynamics during Modified Blalock–Taussig Shunt Operation, Assessed Using FSI Simulation
Source: Materials (Basel). 2022 Apr 7;15(8):2719. doi: 10.3390/ma15082719 (PMC9026531; doi:10.3390/ma15082719)
Supplement: Supplementary file 1 [file materials-15-02719-s001.zip › materials-1493831-supplementary.pdf]

Supplementary Materials

The Effects of the Mechanical Properties of Vascular Grafts and an Anisotropic Hyperelastic Aortic Model on Local Hemodynamics during Modified Blalock–Taussig Shunt Operation, Assessed Using FSI Simulation

Alex G. Kuchumov [1,*], Aleksandr Khairulin [1], Marina Shmurak [1], Artem Porodikov [2] and Andrey Merzlyakov [3]

[1] Department of Computational Mathematics, Mechanics, and Biomechanics, Faculty of Applied Mathematics and Mechanics, Perm National Research Polytechnic University, 614990 Perm, Russia; s.xayrulin@mail.ru (A.K.); shmurak2007@yandex.ru (M.S.)
[2] Federal Center of Cardiovascular Surgery, 614990 Perm, Russia; porodickov.a@yandex.ru
[3] Department of Continuum Mechanics and Computing Technologies, Faculty of Mechanics and Mathematics, Perm State National Research University, 614990 Perm, Russia; merzlyakov@psu.ru
* Correspondence: kychymov@inbox.ru; Tel.: +7-342-2-39-17-02

Introduction

The mechanical properties of arterial wall are mainly characterized by a anatomical structure and physiological behavior. Arterial wall exhibits strongly nonlinear and anisotropic stress-strain response at finite strains. Hyperelastic isotropic and anisotropic models to describe the specific response of arterial walls have been developed throughout the last years [1].

Fluid-structure interaction simulation for explanation of effects occuring in the arterial wall during cardiac cycles are extremly important. However, there is still lack of works describing possibilities and limitations of adoption isotropic and anisotropic models to evaluate arterial wall response. Recently, just several papers have been published [2–5]. Some of them were focused on numerical methods aspects. Some papers were intended to show the discrepancies on the complex geometries. Different models were discussed there. Nevertheless, comparison between anisotropic Holzapfel-Gasser-Ogden and isotropic Ogden model in the tube was not made.

The given report is intended to show the difference between adoption of mentioned above models for blood flow fluid structure interaction simulations in the straight tube.

Problem Formulation

The effect of anisotropy on hemodynamics was tested using the example of a straight vessel. For this purpose, the problem of blood flow in a cylindrical region was solved. The geometric model of the fluid zone is a cylinder with a diameter of 10 mm and a length of 100 mm. The solid zone geometric model is a tube with an inner diameter of 10 mm, a wall thickness of 1 mm, and a length of 100 mm.

The mathematical problem formulation and the inlet boundary conditions (velocity profile during systolic and diastolic phases of the left ventricle) were used the same as in the main text. A constant pressure value P = 100 mm Hg was applied at the outlet.

Blood is assumed to be a Newtonian fluid. The blood density is equal to ρ = 1060 kg/m^3, the dynamic viscosity is constant and equal to μ=0.0035 Pa·s.

The results obtained for two modifications of the model were compared: the first model the vessel walls isotropy; the second model the vessel walls anisotropy. The calculations were performed in the Ansys Workbench software (Cannonsburg, USA). 2-way fluid-solid interaction problem of blood flow in the "straight vessel" was solved.

The parameters of computational mesh of the fluid domain and the solid domain are presented in Table S1 (Figure S1).

Table S1. Parameters of computational mesh.

Body Sizing, mm	Fluid Zone				Solid Zone	
	Inflation			Number of Elements	Body Sizing, mm	Number of Elements
	Transition Ratio	Maximum Layers	Growth Ratio			
1	0.3	7	1.3	99 417	1	7674

Table S2. Mechanical parameters for aorta and shunt used in the study.

Isotropic Hyperelastic Material	Anisotropic Hyperelastic Material
Ogden model:	Holzapfel–Gasser–Ogden model:
$\mu_1 = 1.274$ MPa	$\mu_1 = 2.363$ MPa
$\mu_2 = -1.211$ MPa	$\mu_2 = 0.839$ MPa
$\alpha_1 = 24.074$	$\alpha_1 = 0.6$
$\alpha_2 = 24.073$	$d = 0.001$ MPa^{-1}

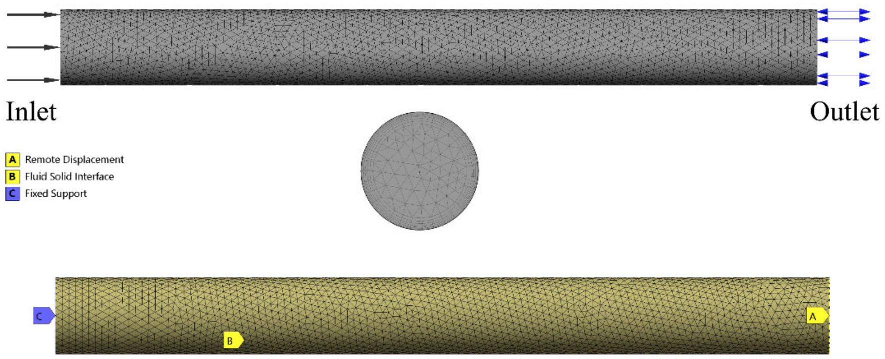

Figure S1. Meshes and boundary conditions of the "straight vessel": upper–boundary conditions and fluid mesh, lower–solid mesh.

Results of FSI Simulation of the Blood Flow

As a result of solving the problem, distributions of hemodynamic parameters were obtained, such as: blood flow velocity, pressure, stress and displacements. The most important results from the hemodynamic point of view were obtained at the moment of time t = 0.09 s, corresponding to the maximum value of the blood flow velocity. The velocity distributions for the isotropic and anisotropic models are practically the same and the pressure distribution at the peak moment of the systole also differs little for this models. The distribution of displacements and stresses for the isotropic and anisotropic models differs in heterogeneity and magnitude (Figures S2 and S3).

The stress and displacement for the isotropic model are uniformly distributed in the computation domain in comparison the anisotropic model. The stress values for the anisotropic model are larger than for the isotropic model, and the displacement values of the isotropic model, on the contrary, significantly exceed similar values for the anisotropic model.

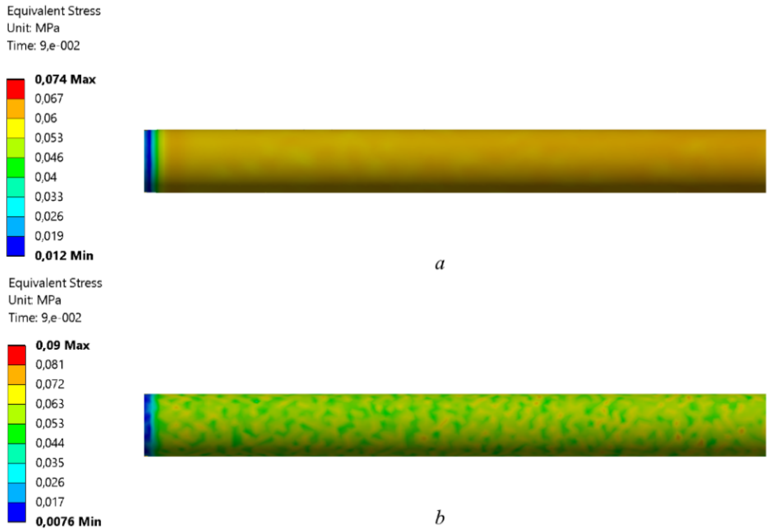

Figure S2. Stress distribution at time t = 0.09 s: (**a**) isotropic properties of the vessel, (**b**) anisotropic properties of the vessel.

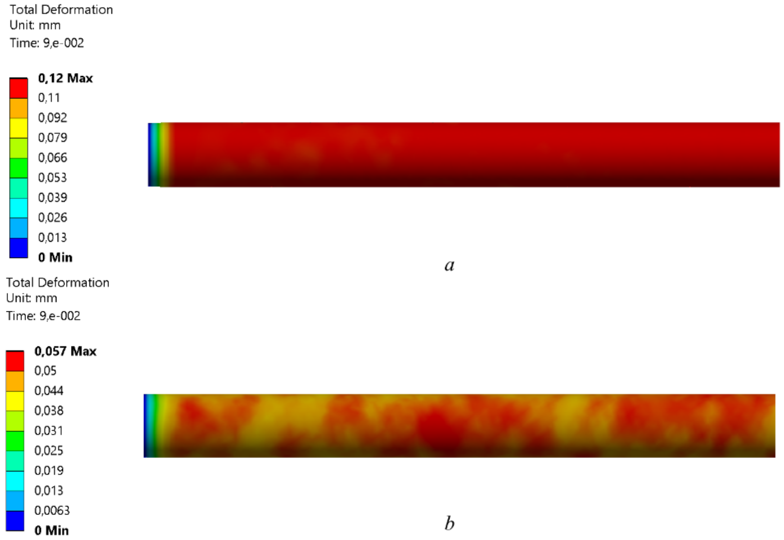

Figure S3. Displacements distribution at time t = 0.09 s: (**a**) isotropic properties of the vessel, (**b**) anisotropic properties of the vessel.

It was found that the estimated displacement in the isotropic model exceed the displacements of the anisotropic model by almost 2 times the maximum values of displacement and stresses. The analysis of the maximum values of the Von Mises stress showed an increase of 20% for the anisotropic model compared to the isotropic one (Figure S4). The maximum values of the wall shear stress for the models are the same.

Conclusions

The distribution of hemodynamic parameters in the anisotropic and isotropic models of vessel material has the same distribution pattern. The numerical values are also the same. The differences between the anisotropic and isotropic properties of vessel are noticeable only when analyzing the stress-strain state. Namely, in displacements and stresses arising in the "straight vessel". Thus, it can be concluded that the anisotropic properties of the wall vessel play an important role in modeling of blood flow.

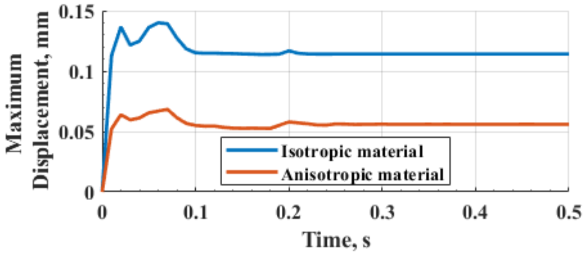

a

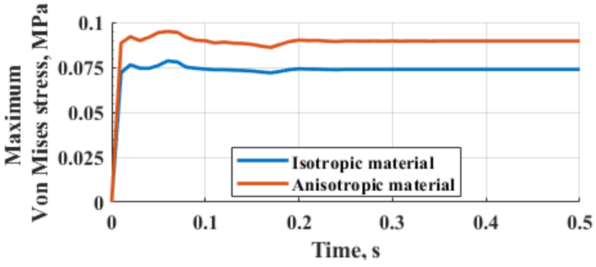

b

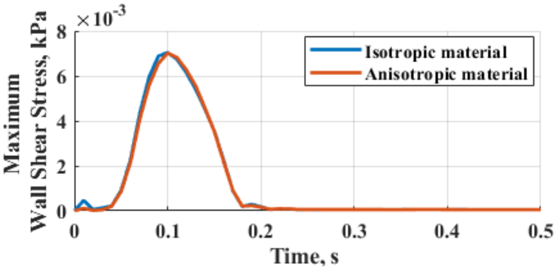

c

Figure S4. Comparison of parameters: (**a**) maximum values of displacements, (**b**) maximum Von Mises stress values, (**c**) maximum wall shear stress values.

References

1. Schmidt, T.; Pandya, D.; Balzani, D. Influence of isotropic and anisotropic material models on the mechanical response in arterial walls as a result of supra-physiological loadings. *Mech. Res. Commun*. **2015**, *64*, 29–37. https://doi.org/10.1016/j.mechrescom.2014.12.008.
2. Weizsacker, H.W.; Pinto, J.G. Isotropy and anisotropy of the arterial wall. *J. Biomech*. **1988**, *21*, 477–487. https://doi.org/10.1016/0021-9290(88)90240-0.
3. Jayendiran, R.; Nour, B.; Ruimi, A. A fluid–structure interaction analysis of anisotropic Dacron fabric used for aortic replacement. *J. Fluids Struct*. **2020**, *97*, 103108. https://doi.org/10.1016/j.jfluidstructs.2020.103108.
4. Balzani, D.; Deparis, S.; Fausten, S.; Forti, D.; Heinlein, A.; Klawonn, A.; Quarteroni, A.; Rheinbach, O.; Schröder, J. Numerical modeling of fluid-structure interaction in arteries with anisotropic polyconvex hyperelastic and anisotropic viscoelastic material models at finite strains. *Int. J. Numer. Method. Biomed.* Eng. **2016**, *32*, e02756. https://doi.org/10.1002/cnm.2756.
5. Tricerri, P.; Dedè, L.; Deparis, S.; Quarteroni, A.; Robertson, A.M.; Sequeira, A. Fluid-structure interaction simulations of cerebral arteries modeled by isotropic and anisotropic constitutive laws. *Comput. Mech.* **2015**, *55*, 479–498. https://doi.org/10.1007/s00466-014-1117-y.
